# Estimation of the prevalence of anxiety during the COVID-19 pandemic: A meta-analysis of meta-analyses

**DOI:** 10.1186/s12889-024-19729-7

**Published:** 2024-10-15

**Authors:** Mostafa Amini-Rarani, Saber Azami-Aghdash, Haleh Mousavi Isfahani, Mohammad Mohseni

**Affiliations:** 1 Social Determinants of Health Research Center, Isfahan University of Medical Sciences, Isfahan, Iran; 2 Tabriz Health Services Management Research Center, Tabriz University of Medical Sciences, Tabriz, Iran; 3 Health Management and Economics Research Center, Isfahan University of Medical Sciences, Isfahan, Iran

**Keywords:** Anxiety, Prevalence, COVID-19, Pandemic, Meta-analysis

## Abstract

**Background:**

Pandemics such as COVID-19, can lead to psychiatric symptoms like anxiety. It seems that meta-analysis of meta-analysis studies can provide more comprehensive information regarding the needs for post-COVID-19 services. Therefore, this umbrella review and meta-analysis of meta-analyses aimed to estimate the precise prevalence of anxiety during the COVID-19 pandemic.

**Methods:**

PubMed, Scopus, and Web of Science were searched for published meta-analyses using relevant keywords, such as Anxiety, Prevalence, COVID-19, and Meta-analysis up to November 1, 2023. Google Scholar, reference check, citation check, and grey literature were manually searched. A random-effect model was used for the analysis. All analyses were conducted using STATA: 17.

**Results:**

Out of the 4263 records, Finally, 75 meta-analyses were included. The overall prevalence of anxiety was 30.4% [95% CI: 29–31.8] with a high heterogeneity (I^2^: 86.76%). The highest prevalence of anxiety according to population type was 41.3% in patients and then in students (30.8), pregnant women (30.6%), and health care workers (30.5%). The Result of meta-regression showed that “Time” (based on the time between the start of COVID-19 and the last search date in articles) was not a significant predictor of the prevalence of anxiety (R Coefficient = 0.000149, *P* = 0.61).

**Conclusions:**

Considering the prevalence of anxiety among patients, students, pregnant women, and healthcare workers, special attention should be paid to these groups in case of the re-occurrence of COVID-19 or occurrence of other pandemics. As quarantine due to pandemics causes reduced social interactions, reduced income, and increased worry about severe illness and death, there is a need for large-scale mobilization of political measures.

**Supplementary Information:**

The online version contains supplementary material available at 10.1186/s12889-024-19729-7.

## Background

Emergence, re-emergence and epidemics are issues that affect the world in various types [1]. Responses to pandemics affect the provision of health care all around the world [2, 3]. Countries apply local or national quarantines to control the incidence of diseases. This has an impact not only on people's daily lives, but also on healthcare systems [4]. The last epidemic in these years was COVID-19, an infectious disease caused by the new corona Virus. The epidemic began at the end of December 2019 and developed into a pandemic [5].

The COVID-19 pandemic led to mass casualties and an economic crisis in many countries and, despite that the disease can be mild to very severe respiratory disease, caused huge death toll in many countries [6, 7]. A strategic goal in the fight against COVID-19 was to limit the consequences for all countries and prevent the global spread of misinformation [8, 9]. In addition to the socio-economic consequences, some individual problems may occur in pandemics. Evidence shows that some people can show psychiatric symptoms such as anxiety, psychological trauma, suicide and panic during epidemics such as COVID-19 [10].

People and especially healthcare workers (HCWs) may feel pressured by issues such as limited access to vaccines, excess workload of healthcare systems, restrictions and quarantines. Some restrictions can lead to reduced social interactions, stress, increased feeling of loneliness and unemployment [11, 12]. In addition, mandatory quarantines due to the diseases lead to the increase in mental problems. One of these problems is anxiety. Anxiety is defined as “apprehensive uneasiness or nervousness usually over an impending or anticipated ill” [13]. Anxiety is usually divided into four categories: mild anxiety, moderate anxiety, severe anxiety and panic level anxiety [14]. A study in 16 countries showed that participants had high levels of anxiety during the COVID-19 pandemic [15]. Another study on 50,000 people in Britain reported that the prevalence of mental distress increased significantly from 19% in 2018 to 27% in April 2020, just one month after COVID-19 quarantine [16].

Due to the increasing prevalence of mental disorders in the COVID-19 pandemic in recent years, many researchers have conducted research on this topic area, and then systematic reviews and meta-analyses were published to summarize the results of these studies. Despite the usefulness of meta-analyses, it seems that there are some controversies in the results of these studies. On the other hand, the large number of meta-analysis papers makes it difficult for healthcare managers and policy makers to select the appropriate paper to apply in their decisions. A meta-analysis of the available meta-analyses can reduce controversy and provide a comprehensive and reliable picture of the evidence on this issue. Therefore, the aim of this umbrella review was to estimate the precise prevalence of anxiety during the COVID-19 pandemic. Findings of this study, by providing precise epidemiologic information, also can be helpful for post-COVID-19 service planning.

## Methods

This systematic review and meta-analysis was conducted in 2024 and followed the Preferred Reporting Items for Systematic Reviews and Meta-Analyses (PRISMA) [17]. All the procedures performed in this study followed the ethical standards of the institutional and national research committees. Institutional review board approval was obtained from the Research Ethics Committee of Isfahan University of Medical Sciences (Ethics code: IR.MUI.NUREMA.REC.1403.025). The protocol for this review was registered with the PROSPERO International Prospective Register of Systematic Reviews (CRD42024500045).

### Search strategy

A comprehensive search of electronic databases (PubMed, Scopus, and Web of Science) was conducted by two independent reviewers using the relevant MeSH keywords up to November 1, 2023. The search terms were derived from previous reviews and consultations with a specialist librarian. The search included the following keywords: “Anxiety”, “Mental health”, “Psychological distress”, “Psychological disorders”, “Mental disorders”, “Psychological impact”, “Prevalence”, “COVID-19”, “Coronavirus”, “Pandemic”, “Meta-analysis” and other related keywords (Supplementary material 1. Search strategy). The reference lists of the included studies and hand-searching through Google Scholar were also used to search for additional studies. Endnote X5 was used to retrieve searches and remove duplicat citations. Two authors (MM and HMI) independently screened the titles and abstracts of the studies, and any disagreements were resolved by discussion with a third author (MA).

### Inclusion and exclusion criteria

All meta-analysis studies published globally in English reporting the prevalence of anxiety during the COVID-19 era were included in this study.

Studies were excluded if they (1) were not published in the English language, (2) were not a meta-analysis study, (3) were published as an editorial, communication, or brief report, (4) were not peer-reviewed, (5) full-text was unobtainable, and (6) were published with poor reporting quality (a score of less than 5 out of 11 in reporting quality assessment).

### Study selection

All stages of article selection and screening were independently performed by two authors. Any disagreement was resolved through discussion, and if necessary, the disputed cases were referred to a third person who had more information and experience. First, the title of all articles were reviewed, and articles that were not compatible with the study objectives were excluded. In the next steps, the abstract and full text of the articles were studied to identify and exclude studies that met the exclusion criteria and those with a weak association with the study aims. Endnote X5 software was used to organize, study the titles and abstracts and identify duplicates. The PRISMA 2020 flowchart was used to report the results of the study selection and screening process.

### Quality assessment

The reporting quality of all articles in full-text screening was independently assessed by two reviewers using the tool for assessment of multiple systematic reviews (AMSTAR) [18]. The responses associated with each item are indicated as "Yes", "No", “Cannot be answered”, or “non-applicable” in this tool. The answer of “Yes” was given a score of 1, and the answers of “No”, “Cannot be answered”, or “non-applicable” were given a score of 0. According to this tool, articles with a score of 1–4, 5–8, and 9–11 are rated as “low quality”, “medium quality”, and “high quality”. Two reviewers’ agreement gave the final assessment score for each article. Discrepancies were resolved by consensus or consultation with a third reviewer.

### Data extraction

Two independent reviewers extracted data from the included studies using a standardized data extraction form. The following data were extracted: sample size, number of included articles in MA, total population reviewed in MA, last date of search, time lag between the start of COVID-19 and the last date of search, prevalence rates of anxiety, severity, and quality score. Consensus or consultation with a third reviewer resolved any discrepancies in data extraction.

### Data analysis

The random effects model was used to perform meta-analysis. The model generalizes findings by assuming that included studies are randomly selected from a larger population [19]. All analyses were conducted using STATA: 17. Forest plot was used to report results. The sample size is shown in the forest plot by the size of each square. Lines on each side of the square show Confidence Interval (CI). I^2^ statistics were used to evaluate the heterogeneity of studies, with cut-off points of low (I^2^ < 50%), medium (I^2^ 50–74%), and high heterogeneity (I^2^ > 75%) [20]. Meta-regression was carried out based on the times between the start of COVID-19 (15/12/2019) and the last search date in articles (week). Publication bias was assessed using funnel plots and Egger's test. Trim and fill analysis was conducted via a linear estimator to test for publication bias.

### Sensitivity analysis

Excluding each study one by one from the analysis did not substantially change the pooled prevalence of anxiety. This indicated that no single study had a disproportional impact on the overall prevalence.

### Subgroup analysis

Sub-group analysis was carried out based on the population type (general population, HCWs, health-care students, patients, and pregnant women, specific groups such as police and teachers, non-medical-student, children/adolescents), gender (male, female), and severity of anxiety (mild, moderate, severe).

## Results

**Fig. 1:**
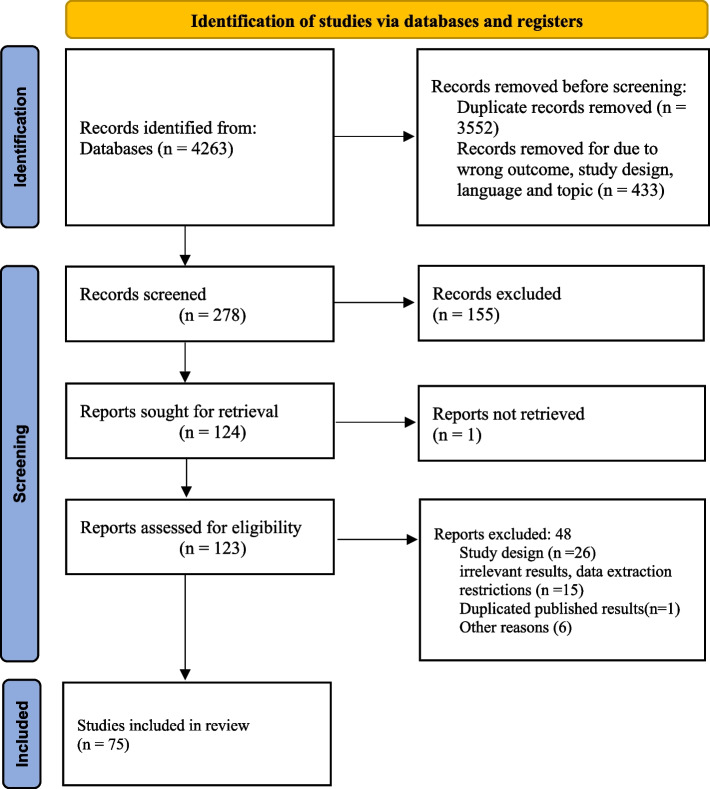
PRISMA flowchart selected studies for review

Out of the 4263 records, 3552 articles were excluded due to database duplication. In the next phase, abstracts and titles were reviewed, and 155 articles were excluded in screening titles and abstracts. In the next phase, 48 articles were excluded in the full-text screening. Finally, 75 meta-analyses were included (Fig. 1).

The highest number of published articles was related to 2021 (47.5%). The number of articles in 2022, 2020, and 2023 was about 31, 11, and 10%, respectively. The most investigated group in all articles was related to HCWs (about 44%) and then the general population (about 24%). After that, there were patients (7.2%) and pregnant women (7.2%) groups. The groups of students (6%), healthcare students (5%), children/adolescents (4%), and specific group (2%) were also in the next categories, respectively. The shortest time to examine the prevalence of anxiety in individuals was about 7.5 weeks, and the longest time was about 130 weeks after the start of COVID-19. The characteristics and results of the included meta-analyses are demonstrated in Supplementary material 2.

### The overall prevalence of anxiety and subgroup analysis

**Fig. 2:**
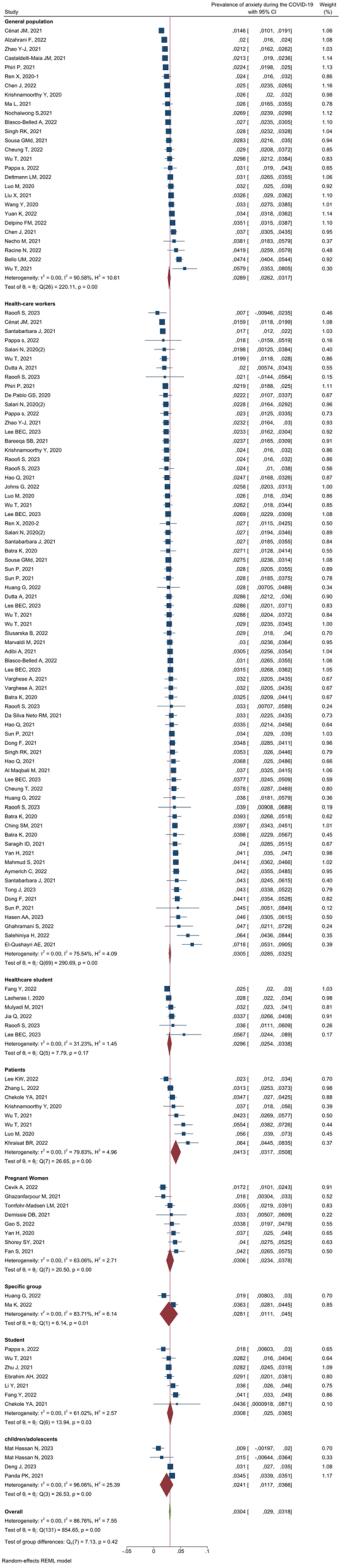
Prevalence of anxiety during the COVID-19

**Fig. 3:**
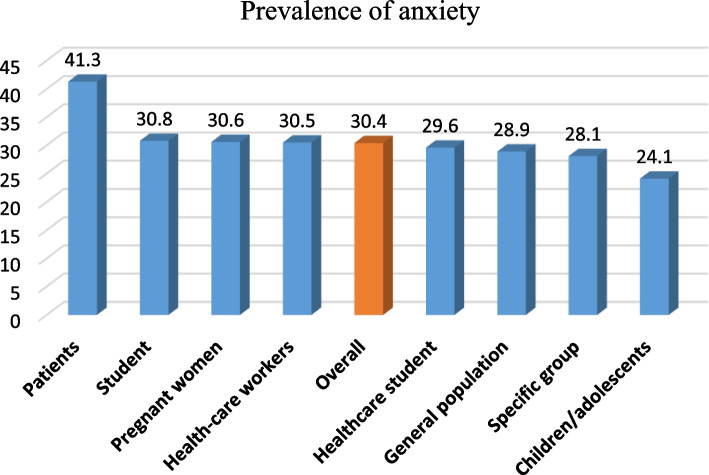
Prevalence of anxiety during the COVID-19 according to groups

**Fig. 4:**
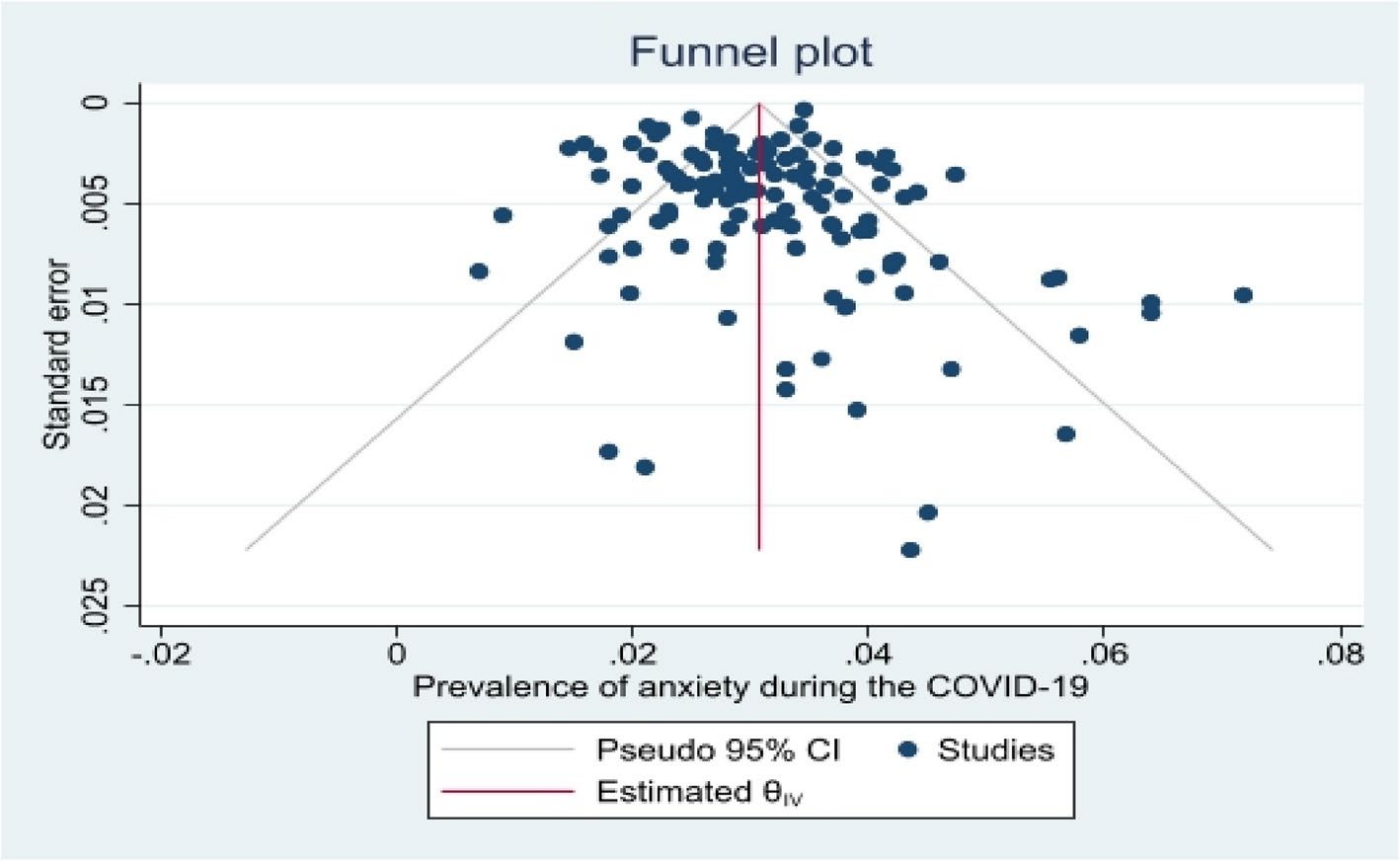
Funnel plot to evaluate the possibility of publication bias

Meta-analysis results showed that the overall prevalence of anxiety was 30.4% [29%–31.8% with 95% CI] (Fig. 2). As shown in Fig. 3, the highest prevalence of anxiety according to population type was 41.3% [31.7–50.8] in patients and then in students (30.8% [25–36.5]), pregnant women (30.6% [23.4–37.8]) and HCWs (30.5% [28.5–32.5]). Heterogeneity assessment results showed high heterogeneity (I^2^: 86.76%) in the results of the studies (Table 1). The results of assessing the potential for publication bias showed a high possibility of publication bias in the findings (Table 1 and Fig. 4). Furthermore, the Trim and Fill test results showed that 23 studies are possibly missing and that with the imputation of these studies and their effect, overall prevalence decreases to 28 [95% CI: 26–30].

**Table 1:** Detailed information about overall prevalence, population type, gender, and severity during the COVID-19 era

Variable	Number of Input	Prevalence%[95% CI]	Heterogeneity (I^2^%)	Publication bias- Egger test (P-value)	Trim and fill test (Observed + imputed)
**Total**	132	30.4 [29–31.8]	86.76	0.0001	30.4% [29–31.8]
General population	27	28.9 [26.2–31.7]	90.58		
Health-care workers	70	30.5[28.5–32.5]	75.54		
Health-care students	6	29.6[25.40–33.8]	31.23		
Patients	8	41.3[31.7–50.8]	79.83		
Pregnant Women	8	30.6[23.4–37.8]	63.06		
Specific group	2	28.1[11.1–45]	83.71		
Student	7	30.8[25–36.5]	61.02		
Children/adolescents	4	24.1[11.7–36.6]	96.06		
**Gender**	23	31.3[26.9–35.8]	84.80	0.83	N/A
Male	11	29.6[23–36.2]	85.37		
Female	12	32.9[26.7–39.2]	83.77		
**Severity**		18.4[13.2–23.5]	98.32	0.0058	23 [18-28]
Mild	9	29.5[17.7–41.3]	97.14		
Moderate	9	18.2[13.8–22.7]	87.34		
Severe	9	7.67[5.69–9.65]	79.62		

*CI *Confidence Interval

### Gender

According to the gender variable (Table 2), the overall estimated prevalence of anxiety in females (32.9% [26.7–39.2]) was more than in males (29.6% [23–36.2]).

**Table 2:**
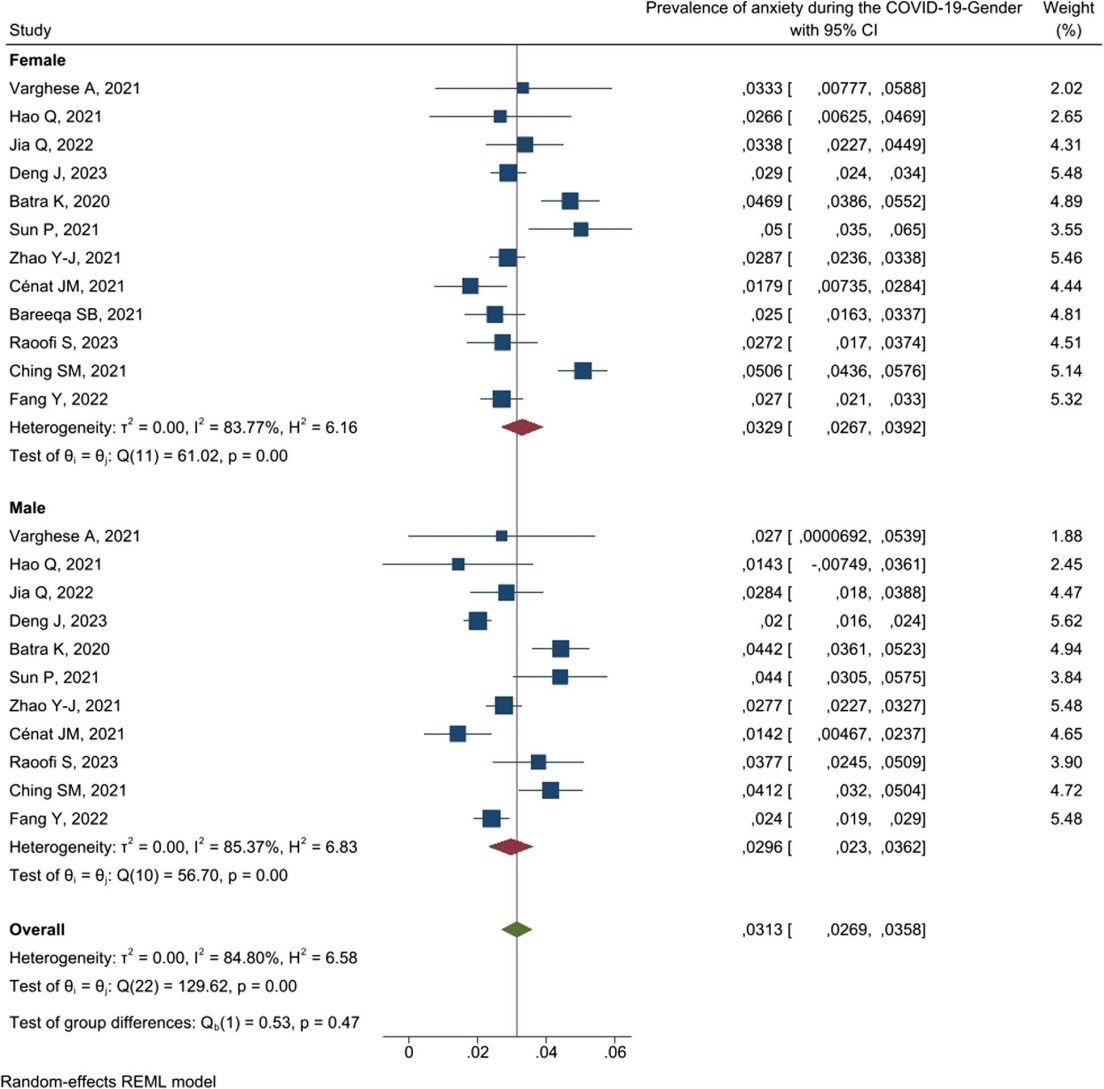
Prevalence of anxiety according to gender [21–32]

### Severity

In terms of severity of anxiety (Table 3), the highest percentage was related to mild, moderate, and severe, with 29.5[17.7–41.3], 18.2[13.8–22.7], and 7.67% [5.69–9.65], respectively.

**Table 3:**
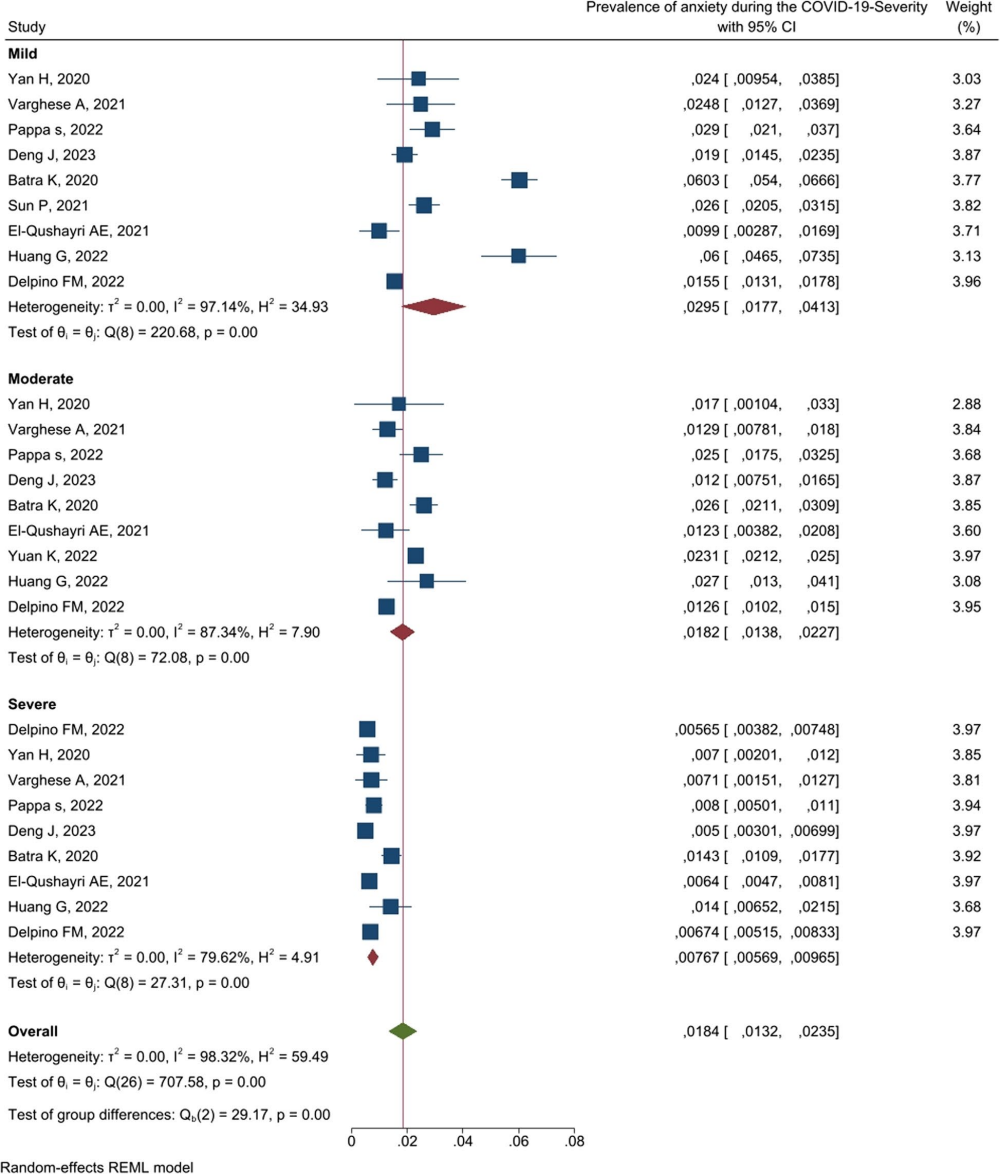
Prevalence of anxiety according to severity [21, 22, 26, 30, 33–38]

### Meta-regression

Meta-regression results with a random model, based on the times lag between the start of COVID-19 and the last date of search in articles (week), showed that every week passed of COVID causes the prevalence of anxiety to increase by almost 0.000149% [95% CI: -0.000425, 0.000722], P-value: 0.61. These results showed that “Time” was not a significant predictor of an increase in the prevalence of anxiety.

## Discussion

The results of the present meta-analysis showed that the overall prevalence of anxiety was 30.4%. The highest prevalence was in the group of patients (41.3%) and then in students (30.8%), pregnant women (30.6%), and HCWs (30.5%). Almost all studies have shown that the prevalence of anxiety symptoms was high, and these symptoms increased during the pandemic compared to the period prior to it [39]. The World Health Organization (WHO) report also shows that the global prevalence of anxiety increased by 25% in the first year of the pandemic [40].

The results of studies on other mental health problems show that in addition to anxiety, problems such as depression, post-traumatic stress disorder (PTSD), insomnia, fear, and sleep problems have also increased as a result of the COVID-19 pandemic. According to the results of a meta-analysis study, the prevalence of depression during the COVID-19 pandemic in the Asia Pacific region was estimated at 34% [41]. In other studies, this disorder has been reported to be about 24% for general population [42] and 40% [43] for HCWs. The results of other meta-analyses also showed that the prevalence of PTSD was about 11, 16 and 30% [44–46], sleep problems 45.1 [47] and 35.7% [48], insomnia 52.6% [49] and fear 13.1% [50]. These results show that the prevalence of anxiety is also almost similar to other mental health problems during the COVID-19 period. Given that anxiety is a significant social and mental health issue during pandemics such as COVID-19, especially in low and middle-income countries [51], there is a need to pay more attention to anxiety and to solve it at different times, including before, during and after disease, especially in economically weaker countries.

Based on the other findings of the present study, the highest prevalence of anxiety was observed in the patient group (41.3%). Given the lack of proper treatment and vaccines during the early months of the pandemic, this finding seems reasonable. The high prevalence of mortality, especially in some countries and individuals with chronic diseases, could be the cause of increasing anxiety in people. By December 31, 2020, COVID-19 was declared a Public Health Emergency, with an official death toll of 1,813,188. However, preliminary estimates suggest that the total number of global deaths attributable to the COVID-19 pandemic in 2020 was at least 3 million [52]. Strategies such as listening to their concerns, explaining the disease, appropriate patient care, adequate rest, and appropriate medications can be used to reduce the patients' anxiety.

Another finding of the current study was that the highest prevalence of anxiety after the patient group was related to students (30.8%). Anxiety in students is influenced by various factors such as gender, fear of infection, academic performance, social support, coping strategies, and knowledge about the pandemic [53, 54]. Given that the pandemic has caused unprecedented anxiety among students, it is necessary to take action to address these issues [55]. To reduce students' anxiety, strategies such as self-care training, communication skills, increasing resilience, and training the parents and teachers can be used [56].

According to other results, the highest number of published articles was related to 2021 (47.5%), and the most investigated group in all articles was related to HCWs (44%). This issue may be due to the increased interaction between HCWs with patients and suspected individuals compared to the rest of society. Due to these high interactions and workload, the structure of the healthcare system changed rapidly during the epidemic, and it was observed that the mental and physical situation of frontline health workers reached levels of severe clinical and psychological concern [57, 58].

Given the prevalence of pandemics such as COVID-19 in all countries and groups, some interventions can be performed to prevent and reduce anxiety. Examples include governmental programs [59], social support [60], education [61], physical interventions [62], self-care [63] and psychotherapy [64]. Final results showed that the time passed of COVID-19 was not a significant predictor of the prevalence of anxiety. This indicates that over time, probably due to finding appropriate treatments and vaccines, sensitivity to the disease has decreased, and the level of anxiety has not increased further after a certain period.

This study has some limitations. First, the search in this study and most included meta-analyses was limited to the English language. Second, the researchers wanted to combine the findings of published meta-analyses, so they did not exclude duplicate original studies included in the meta-analyses. Another limitation of the findings of the current study was that the heterogeneity test showed the possibility of publication bias in the included studies. However, the Trim and Fill test showed that the impact of such bias might not be considerable.

## Conclusion

This meta-analysis showed that the prevalence of anxiety was high during the COVID-19 pandemic, particularly among patients, students, pregnant women, and healthcare workers. These groups should receive more attention in cases of re-occurrence of the COVID-19 pandemic or occurrence of similar pandemics. Preventive and therapeutic measures for the management of anxiety may be implemented by policymakers for all population groups, especially for groups mentioned above. Considering the quarantine that results in reduced social interactions, reduced income, and increased worry about severe illness and death, mobilization of political attention is required to address the mental health of the population.

## Supplementary Information

Supplementary material 1. Complete search strategy for the databases.

Supplementary material 2. Extraction Table [21–38, 65–127].

## Data Availability

Data is provided within the manuscript or supplementary information files.
