# Supplementary material for: Estimation of the prevalence of anxiety during the COVID-19 pandemic: A meta-analysis of meta-analyses
Source: BMC Public Health. 2024 Oct 15;24:2831. doi: 10.1186/s12889-024-19729-7 (PMC11476206; doi:10.1186/s12889-024-19729-7)
Supplement: Supplementary file 1 — Supplementary material 1. Complete search strategy for the databases. [file 12889_2024_19729_MOESM1_ESM.docx]

| **Supplementary material 1.** Complete search strategy for the databases | |
| --- | --- |
| **PubMed** | |
| 1454 | (Psychological Disorders[Title]) OR (Mental Disorders[Title])) OR (psychological impact[Title])) OR (psychological consequences[Title])) OR (mental health[Title])) OR (anxiety[Title])) OR (Psychological[Title]) AND(covid[Title]) OR (2019 novel coronavirus[Title])) OR (COVID19[Title])) OR (COVID-19[Title])) OR (COVID 2019[Title])) OR (2019-novel CoV[Title])) OR (SARS-cov-2[Title])) OR (SARS-CoV2[Title])) OR (SARSCoV2[Title])) OR (SARSCoV-2[Title])) OR (severe acute respiratory syndrome coronavirus 2[Title])) OR (2019-ncov[Title])) OR (coronavirus disease 2019[Title])) OR (coronavirus disease-19[Title])) OR (2019ncov[Title])) OR (SARS coronavirus2[Title])) OR (severe acute respiratory syndrome coronavirus 2[Title])) OR (COVID-19[Title]) AND(Meta analysis[Title]) OR (Meta-analysis[Title]) OR (Systematic Review[Title]) |
| **Scopus** | |
| 1936 | ("Psychological Disorders" OR "Mental Disorders OR "psychological impact" OR "psychological consequences" OR "mental health" OR anxiety OR "Psychologica") AND covid OR "2019 novel coronavirus" OR COVID19 OR COVID-19 OR "COVID 2019" OR "2019-novel CoV" OR SARS-cov-2 OR SARS-CoV2 OR SARSCoV2 ORSARSCoV-2" OR "severe acute respiratory syndrome coronavirus 2" OR 2019-ncov OR "coronavirus disease 2019" OR "coronavirus disease-19" OR 2019ncov OR "SARS coronavirus2" OR "severe acute respiratory syndrome coronavirus 2" OR COVID-19) AND ("Meta analysis" OR "Meta-analysis" OR "Systematic Review") AND  (LIMIT-TO ( LANGUAGE ,  "english" ) )  AND  LIMIT-TO ( DOCTYPE ,  "re" ) )  AND  ( LIMIT-TO ( SUBJAREA ,  "medi" ) |
| **Web of Science** | |
| 873 | TS=("Psychological Disorders" OR "Mental Disorders" OR "psychological impactv" OR "psychological consequences" OR "mental health" OR anxiety OR Psychological) AND TS=(covid OR "2019 novel coronavirus" OR COVID19 OR COVID-19 OR "COVID 2019" OR "2019-novel CoV" OR SARS-cov-2 OR SARS-CoV2 OR SARSCoV2 OR SARSCoV-2 OR "severe acute respiratory syndrome coronavirus 2" OR 2019-ncov OR "coronavirus disease 2019" OR "coronavirus disease-19" OR 2019ncov OR "SARS coronavirus2" OR "severe acute respiratory syndrome coronavirus 2" OR COVID-19) AND TS=("Meta analysis" OR Meta-analysis OR "Systematic Review") |
