# Supplementary material for: Estimation of the prevalence of anxiety during the COVID-19 pandemic: A meta-analysis of meta-analyses
Source: BMC Public Health. 2024 Oct 15;24:2831. doi: 10.1186/s12889-024-19729-7 (PMC11476206; doi:10.1186/s12889-024-19729-7)
Supplement: Supplementary file 2 — Supplementary material 2. Extraction Table [21–38, 65–127]. [file 12889_2024_19729_MOESM2_ESM.docx]

| **Supplementary material 2:** Extraction Table [[21](https://bmcpublichealth.biomedcentral.com/articles/10.1186/s12889-024-19729-7#ref-CR21)-[38](https://bmcpublichealth.biomedcentral.com/articles/10.1186/s12889-024-19729-7#ref-CR38), [65](https://bmcpublichealth.biomedcentral.com/articles/10.1186/s12889-024-19729-7#ref-CR65)-[127](https://bmcpublichealth.biomedcentral.com/articles/10.1186/s12889-024-19729-7#ref-CR127)] | | | | | | |
| --- | --- | --- | --- | --- | --- | --- |
| **First author, Publication Year** | **Sample** | **Sample Size** | **Last date of search** | **%Prevalence[CI]** | **Subgroup**  %Prevalence[CI] (Sample Size**)** | **Severity**  %Prevalence[CI] (Sample Size**)** |
| Yan H, 2020 | Pregnant and Postpartum Women | 10424 | 19/9/20 | 37[25-49] |  | -Mild:24[11-40] (4905)  -Moderate:17[4-36] (5693)  -Severe:7[3-13] (5693) |
| Ren X, 2020 | GP & Health-care Professionals | 27475 | ../4/20 | 25[0.19–0.32] | -GP:24 [16-32] (21377)  -Health professionals:27[12-43] (6098) |  |
| De Pablo GS, 2020 | Health  Care Workers | 7716 | 15/4/2020 | 0.222[0.127 0.358] |  |  |
| Luo M, 2020 | Medical Staff And GP | 162,639 | 25/5/2020 | 33[28-38] | -GP:32[25-39]  -HCW: 26[18-34]  -Patients or caregiver: 56[39-73] |  |
| Salari N, 2020, 2 | Front-Line HCW | 19768 | ../6/2020 | 25.8[20.5-31.9] | -Hospital staff (non-physicians and nurses): 27[20.1-35.3] (11062)  -Physicians: 19.8[7.1-44.3] (643)  -Nurses: 22.8[17-29.8] (8063) |  |
| Batra K, 2020 | HCW | 51596 | 27/7/2020 | 34.4[29.5-39.7] | -Males: 44.2[36.3-52.5]  -Female: 46.9 [38.6-55.3]  -Nurses: 39.3 [27.5-52.6]  -Doctors: 32.5 [21.9-45.2]  -Frontline: 39.8[24.1–58.0]  -Second-line: 27.1[15.1–43.7] | -Mild: 60.3[53.8–66.4]  -Moderate: 26[21.4–31.3]  -Severe: 14.3 [11.2–18.1] |
| Krishnamoorthy Y, 2020 | GP, HCW And COVID-19 Patients | 96025 | 22/4/2020 | 26[21-31] | -GP:26[20-32]  -HCW:24[16-32]  COVID-19 patients:37[19-57] |  |
| Lasheras I, 2020 | Medical Students | 11710 | 26/8/2020 | 28[22–34] |  |  |
| Singh RK, 2021 | Health Care  Workers And GP | 2217 | 10/10/2020 | 34.1[26.3–42.3] | -HCW: 35.3[26.3–44.9]  -GP: 28.0[23.4–33] |  |
| Sun P, 2021 | HCW | 81,277 | 20/9/2020 | 37[31–42] | **-**Frontline:45[5-85]  -Non-frontline:28[21-36]  -Doctor:28[19-38]  -Nurse:34[26-41]  -Male:44[31-58]  -Female:50[35-65] | -Mild:26[20-31]  -Moderate-severe: 23[19-27] |
| Zhao Y-J, 2021 | GPs And Health Professionals | 45253 | 25/5/2020 | 23.4[19.9 - 27.3] | -GPs:21.2[16.6 -26.7] (20599)  -Health professionals:23.2[17.1 - 30.8] (13020)  -Male: 25.7 [21.0 - 31.1] (9663)  -Female: 28.7 [23.8 - 34.1] (17907) |  |
| Marvaldi M, 2021 | HCW | 51942 | 8/10/2020 | 30[24.2–37.05] |  |  |
| Sousa GMd, 2021 | GP And HCW | 1,074,438 | 2/3/2021 | 27.77[24.47–31.32] | -GP: 28.33[22.1– 35.5]  -HCW: 27.5[23.78–31.55] |  |
| Mahmud S, 2021 | Health Professionals | 147435 | 30/3/2021 | 41.42[36.17–46.54] |  |  |
| Al Maqbali M, 2021 | Nurses | 81561 | 26/10/2020 | 37[32–41] |  |  |
| Cénat JM, 2021 | Affected Populations | 121373 | 12/5/2020 | 15.15[12.29-18.54] | -Citizens: 14.62[10.69;19.69] (84297)  -HCW:15.86[12.22;20.33] (37076)  -Male: 14.19[7.14;26.23]  -Female: 17.87[9.64;30.73] |  |
| Santabarbara J, 2021 | Health Care Professionals | 12131 | 15/9/2020 | 25[21–29] | Nurses:27[20–34] (6513)  Medical doctors: 17[12–22] (5177)  Frontline HCW:43[25–62] (441) |  |
| Fan S, 2021 | Pregnant Women | 7878 | 27/9/2020 | 42[26–57] |  |  |
| Da Silva Neto RM, 2021 | Health Professionals | 7102 | ../4/2020 | 33[24-45] |  |  |
| Adibi A, 2021 | HCW | 21,866 | 30/6/2020 | 30.5 [25.58–35.42] |  |  |
| Bareeqa SB, 2021 | Chinese People | 57311 | ../4/2020 | 21.8[16.9–27.1] | -Frontline HCW: 23.7[16.8-31.3] (10267)  -Female: 25[16.8–34.3] (21391) |  |
| Dong F, 2021 | HCW | 27097 | 7/10/2020 | 34.4[29.5–39.4] | -Frontline HWs: 34.8[28.8–41.4]  -Nurses:44.1[35.4–52.8] |  |
| El-Qushayri AE, 2021 | HCW | 1344 | 15/1/2021 | 71.8[49.4-86.9] |  | -Moderate: 12.3[6.1-23.1]  -Mild: 9.9[4.9-19]  -Severe:6.4[4.9-8.3] |
| Phiri P, 2021 | Patients, GP And Health-care Professionals | 721,244 | 22/1/2021 |  | -Healthcare professionals: 21.9[18.7-25]  -Public: 22.4[19.8-25] |  |
| Dutta A, 2021 | HCW | 23472 | 15/8/20 | 32.5[26.4–39.0] | -Medical (doctors&nusres:28.6 [21.5-36.3]  -Other HCW: 20 [7.5-36.1] |  |
| Varghese A, 2021 | Nurses | 13641 | 5/10/20 | 32[21-44] | -Males:27 [4-58] (206)  -Female: 33.3 [10.4-61.6] (5138) | -Mild:24.8 [13.7-37.9] (3195)  -Moderate:12.9 [8.2-18.4] (3266)  -Severe:7.1 [2.5-13.7] (3442)  -Extreme severe:2 (421) |
| Demissie DB, 2021 | Women Who Are Pregnant and/or Lactating | 16627 | 30/9/20 | 33[5-61] |  |  |
| Saragih ID, 2021 | Health-care  Workers | 33482 | 2/11/20 | 40 [29-52] |  |  |
| Zhu J, 2021 | Post-Secondary Students | 1280466 | 3/5/20 | 28.2[24.6-32.1] |  |  |
| Chen J, 2021 | 12 African Countries | 14847 | 6/2/20 | 37[31-44] |  | -Above mild: 62[56–67] (6174)  -Above moderate:34[29-40](3511)  -Above severe:14[10–20] (4133) |
| Ma L, 2021 | Children And Adolescents | 42435 | ../9/20 | 26[16-35] |  |  |
| Mulyadi M, 2021 | Nursing Students | 9554 | 29/6/21 | 32[24-42] |  |  |
| Hao Q, 2021 | HCW | 10015 | 14/4/20 | 28.6[22.4-36.4] | -Males: 14.3 [4.3-48] (475)  -Female: 26.6 [13.1-53.9] (1444)  -Nurse: 36.8[26.8-50.5] (3902)  -First line:33.5[23.5-47.7] (2002)  -Second line:24.7[18-33.9] (2920) |  |
| Nochaiwong S,2021 | GP | 284813 | 16/6/20 | 26.9[24-30] |  |  |
| Wu T, 2021 | Public | 128,855 | 16/3/20 | 31.9[(27.9–36.0] | -GP: 29.8 [21.5–38.8] (57,898)  -Noninfectious chronic disease patients: 55.4 [37.8–72.3] (185)  -Students: 28.2 [16.8–41.2] (17257)  -Physicians & nurses: 29.0 [23.6–34.7] (50143)  -Other medical staff: 19.9 [12.4–28.6] (2521)  -Quarantined persons: 57.9 [34.4–79.7] (367)  -COVID-19 patients: 42.3 [27.3–58.1] (484)  -Front-line physicians and nurses: 28.8[20.7-37.6] (10429)  -Second-line physicians and nurses: 26.2[18.4-34.8] (19400) |  |
| Liu X, 2021 | Public | 86035 | 1/7/2020 | 32.60[29.10-36.30] |  |  |
| Yan H, 2021 | Medical Staff | 25,343 | 19/4/2020 | 41[35-47] |  |  |
| Li Y, 2021 | College Students | 37912 | ../10/2020 | 36[26-46] |  |  |
| Chekole YA, 2021 | GP | 88543 | ../4/2020 | 33.59[27.21-39.97] | -Students:43.62[11.56-98.80]  -Patients: 34.72[26.95-42.50] |  |
| Tomfohr-Madsen LM, 2021 | Pregnant Women | 42773 | ../2/2021 | 30.5[22.6-39.8] |  |  |
| Panda PK, 2021 | Children/Adolescents | 22996 | 15/8/2020 | 34.5[33.8–35.1] |  |  |
| Ghazanfarpour M, 2021 | Pregnant Women | 17738 |  | 18.7[6–36] |  |  |
| Khraisat BR, 2022 | Eating Disorder Patients | 2444 |  | 64[39-78] |  |  |
| Aymerich C, 2022 | Health Worker’s | 206513 | 1/3/2020 | 42:35–48] |  |  |
| Alzahrani F, 2022 | GP | 212108 | 17/8/2020 | 20[16-24] |  |  |
| Salehiniya H, 2022 | Dentists | 1472 | .../7/2020 | 64[41-82] |  |  |
| Chen J, 2022 | Developing  Countries | 1257838 | 22/9/2021 | 25[24-27] | -Frontline HCWs:27[24-29] (71539)  -General HCWs:25[23-27] (123698)  -GP:23[21-25] (697481)  -Adult students:30[27-32] (796214)  -Medical students:38[32-44] (18890) | -Above mild: 43[41-44] (1213070)  -Above moderate: 21[20-23] (385010)  -Above severe: 8[7-9] (158396) |
| Lee KW, 2022 | People Living With HIV | 938 | 30/4/2021 | 23[12-34.0] |  |  |
| Zhang L, 2022 | Cancer Patients | 27590 | 31/1/2022 | 31.3 [25.4-37.5] |  |  |
| Racine N, 2022 | Mothers Of Young Children | 4811 | 3/3/2021 | 41.9[26.7-58.8] |  |  |
| Jia Q, 2022 | Medical Students | 34285 | 18/8/2021 | 33.7[26.8-41.1] | -Male: 28.4[19.2-40.0]  -Female:33.8[23.6-45.9] |  |
| Pappa s, 2022 | General Adult Population And HCW | 20352 | ../2/2021 | 22[17-27] | -Frontline HCW:23[13-34]  -General HCW:18[12-80]  -GP:31[20-44]  -Student 18[8-32] | -Mild: 29[21-37]  -Moderate: 25[18-33]  -Severe:8[5-11] |
| Ghahramani S, 2022 | Hcw | 53463 | 20/2/2022 | 47[22–74] |  |  |
| Dettmann LM, 2022 | GP | 46158 | 1/9/2021 | 31[26-35] |  |  |
| Blasco-Belled A, 2022 | GP And HCW | 880352 | 6/12/2020 |  | -GP:27[0.23-0.30]  -HCW:31[0.27-0.36] |  |
| Arora T, 2022 | HCW And GP | 97173 | ../4/2020 | 28[21–36] |  |  |
| Yuan K, 2022 | All Population | 727602 | 14/8/2020 | 34 [31.8-36.3] |  | -Moderate: 23.1 [21.2-25.1] (331227) |
| Gao S, 2022 | Postpartum Women | 12493 | 16/6/2021 | 33.8[21.1–49.4] |  |  |
| Raoofi S, 2023 | Hospital Staff | 61551 | ../2/2021 | 26.1[19-34.6] | -Health technicians:39[13-73]  -Medical students:36[15-65]  -Hospital managers: 7[1-34]  -Nurses: 24[17-33]  -Doctors: 24[12-40]  -Support staff: 21[0-90]  -Other hospital workers:33[12-64]  -Women: 37.7[25.4-51.8]  -Men: 27.2[18.2-38.6] |  |
| Lee BEC, 2023 | Hospital HCW | 295578 | 17/2/2022/ | 28.7[26.5-31.0] | -Nurses:31.5[26.9-36.3] (72695)  -Physicians:26.9[23-31] (37443)  -Allied health:23.3[16.5-30.7] (4856)  -Non-medical staff:28.6[20.4-37.5] (3616)  -Support staff:37.7[24.9-51.4] (2819)  -Healthcare student:56.7[22.8-87.5] (343) |  |
| Deng J, 2023 | Children And Adolescents ≤18 Years Of Age | 1241604 |  | 31[27-35] | -Male:20[16-24] (552512)  -Female:29[24-34] (531432) | -Mild:19[15-24] (501614)  -Moderate:12[8-17] (501211)  -Severe:5[3-7] (508785) |
| Tong J, 2023 | Frontline HCW | 17013 | 31/12/2021 | 43[33.8–52.3 |  |  |
| Mat Hassan N, 2023 | Children With Long COVID | 574 | ../5/2022 | 9[1-23] |  |  |
| Aldhamin RA, 2023 | HCW | 7947 | ../4/2021 |  |  | -Moderate to severe anxiety: 34.57[19.73-51.12] |
| Wang Y, 2020 | GP | 207783 | 15/7/2020 | 33[28-39] |  |  |
| Shorey SY, 2021 | Women In The Perinatal Period | 8547 | ../12/2020 | 40[27-52] |  |  |
| Castaldelli-Maia JM, 2021 | Global | 193137 | 29/7/2020 | 21.3[19-23.6] |  |  |
| Necho M, 2021 | General  Population | 78225 |  | 38.12 [18.29-57.96] |  |  |
| Ching SM, 2021 | Health-care  Providers | 99799 | 15/3/2021 | 39.7[34.3–45.1] | -females:50.6[43.5–57.6]  -males:41.2[32.0–50.4] |  |
| Cheung T, 2022 | HCW, GP And Affected Individuals | 27325 | 1/6/20 | 34.8[29.1–40.4] | -HCW:37.8[28.7–46.9]  -GP:29 [20.8–37.2] |  |
| Andhavarapu S, 2022 | HCW | 117,143 | 4/5/2022 | -PTSD: 34[30-39] |  |  |
| Huang G, 2022 | First Responders For Medical Emergencies | 7975 |  | 32[20-44] | **-**Paramedics: 38[20-60]  -EMS personnel: 28[11-53]  -Police: 19[10-32] | -Mild: 60[46-73] (1468)  -Moderate: 27[14–42] (1475)  -Severe: 14[7-22] (1475) |
| Ebrahim AH, 2022 | University  Students | 22357 | 11/9/2020 | 29.1[20.9–39] |  |  |
| Ślusarska B, 2022 | Nurses | 43062 | 18/2/2021 | 29[18-40] (43,062 |  |  |
| Ma K, 2022 | Teachers | 256896 | ../7/2021 | 36.3[28.5–44.9] |  |  |
| Johns G, 2022 | Doctors | 33281 | 3/3/2021 | 25.8[20.4-31.5] |  |  |
| Delpino FM, 2022 | GP | 2424638 | ../6/2021 | 35.12[31.57-38.84] |  | -Mild: 15.45[13.21–17.98] (513,468)  -Moderate: 12.63[10.41-15.26] (531202)  -Severe: 6.74[5.32–8.51] (468276)  -Extremely severe:5.65[4.09–7.75] (111999) |
| Bello UM, 2022 | GP | 31300 | 30/9/2021 | 47.4[40.4–54.4] |  |  |
| Fang Y, 2022 | Student | 2048035 | 10/3/2022 | 28[24-32] | -Male: 0.24 [0.19-0.29]  -Female: 0.27 [0.21-0.33]  -Medical: 0.25 [0.20-0.30]  -Non-medical: 0.41 [0.33-0.49] |  |
| Cevik A, 2022 | Postpartum Women | 77299 | ../5/2021 | 17.2 [0.112–0.255] |  |  |
| Hasen AA, 2023 | Health-care  Professionals | 3703 | ../6/2022 | 46[0.30-0.61] |  |  |
| HCW: Health-care workers  GP: General population | | | | | | |
